# Fluvoxamine alleviates ER stress via induction of Sigma-1 receptor

**DOI:** 10.1038/cddis.2014.301

**Published:** 2014-07-17

**Authors:** T Omi, H Tanimukai, D Kanayama, Y Sakagami, S Tagami, M Okochi, T Morihara, M Sato, K Yanagida, A Kitasyoji, H Hara, K Imaizumi, T Maurice, N Chevallier, S Marchal, M Takeda, T Kudo

**Affiliations:** 1 Department of Psychiatry, Osaka University Graduate School of Medicine, Suita, Osaka, Japan; 2 Department of Psychiatry, Osaka General Medical center, Sumiyoshi-ku, Osaka, Japan; 3 Gifu Pharmaceutical University, Department of Biofunctional Molecules, Gifu, Japan; 4 Department of Biochemistry, Graduate School of Biomedical & Health Sciences Hiroshima University, Hiroshima, Japan; 5 Team II Endogenous Neuroprotection in Neurodegenerative Diseases INSERM U. 710, EPHE, University of Montpellier cc 105, place Eugene Bataillon, Montpellier cedex 5, France; 6 Department of Psychiatry, Osaka University Health Care Center, Toyonaka, Osaka, Japan

## Abstract

We recently demonstrated that endoplasmic reticulum (ER) stress induces sigma-1 receptor (Sig-1R) expression through the PERK pathway, which is one of the cell's responses to ER stress. In addition, it has been demonstrated that induction of Sig-1R can repress cell death signaling. Fluvoxamine (Flv) is a selective serotonin reuptake inhibitor (SSRI) with a high affinity for Sig-1R. In the present study, we show that treatment of neuroblastoma cells with Flv induces Sig-1R expression by increasing ATF4 translation directly, through its own activation, without involvement of the PERK pathway. The Flv-mediated induction of Sig-1R prevents neuronal cell death resulting from ER stress. Moreover, Flv-induced ER stress resistance reduces the infarct area in mice after focal cerebral ischemia. Thus, Flv, which is used frequently in clinical practice, can alleviate ER stress. This suggests that Flv could be a feasible therapy for cerebral diseases caused by ER stress.

Sigma-1 receptor (Sig-1R) is expressed on endoplasmic reticulum (ER) membranes. Several functions have been attributed to Sig-1R, including regulation of ion channels such as Ca2+ and K+ channels, inhibition of Ca2+ influx through the *N*-methyl-D-aspartate (NMDA) receptor, modulation of the release of neurotransmitters such as dopamine, regulation of lipid distribution, cell differentiation, and behavioral sensitization to cocaine and other stimulants.^1^ Recently, Sig-1R was shown to have neuroprotective activity, and several studies have demonstrated that it acts as a molecular chaperone.^2, 3, 4^ Under normal conditions, Sig-1R forms a complex with another molecular chaperone, GRP78/BiP, on the ER membrane. Under ER stress, Sig-1R dissociates from BiP, interacts with IP3 receptors, and stabilizes IP3 receptor structure.^4^

Numerous studies have examined the role of Sig-1R in the pathogenesis of psychiatric diseases. Postmortem analysis has shown that Sig-1R expression is reduced in the brains of schizophrenia patients.^5^ Additionally, Sig-1R knockout mice exhibit symptoms of depression.^6^ Given these observations, it is possible that reduction of Sig-1R is a pathogenic factor in disorders such as schizophrenia and depression. Therefore, numerous synthetic compounds that bind to Sig-1R, including antidepressants and antipsychotic drugs, have been examined as therapeutic targets for these disorders.^7^ However, the results of clinical testing have not been satisfactory.^8^ One possible reason is that the effects of compounds that bind to Sig-1R cannot fully manifest because Sig-1R expression is reduced in the brains of patients with psychiatric diseases. This led us to examine whether compounds capable of inducing Sig-1R expression might be therapeutic in these diseases.

When cells encounter ER stress, Sig-1R expression increases in response to activation of the PERK pathway, which is one of the cellular responses to ER stress.^9^ In addition, the induction of Sig-1R expression can repress cell death signals that accompany ER stress.^9^

Fluvoxamine (Flv) is a selective serotonin reuptake inhibitor (SSRI) that is widely used in clinical practice as an antidepressant. Because Flv is a potent Sig-1R agonist that exhibits a stronger affinity for Sig-1R than for other SSRIs,^10^ we investigated its effect on Sig-1R expression and on the cellular ER stress responses.

## Results

### Flv induces Sig-1R expression

**Figure 1:**
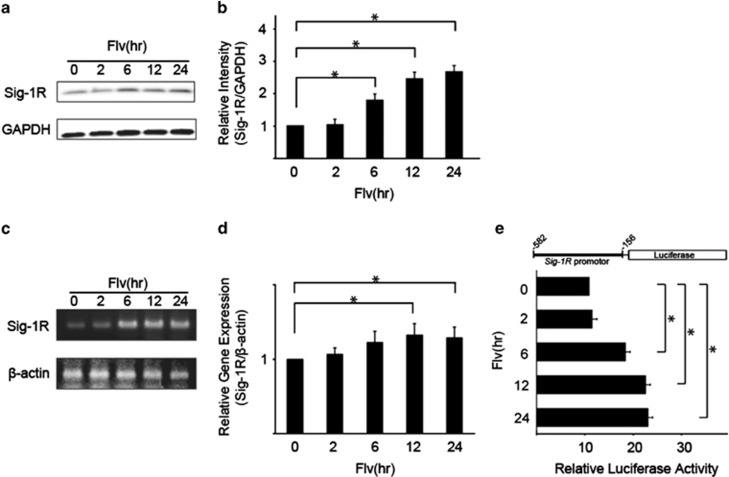
Fluvoxamine (Flv) induces the expression of sigma-1 receptor (Sig-1R). (**a**) Flv (10 *μ*g/ml) was added to Neuro2a cells, and Sig-1R protein and GAPDH protein levels were monitored over the time course (h) shown in the figure. (**b**) The amount of Sig-1R and GAPDH protein in (**a**) was quantified with densitometry. Relative intensity of Sig-1R/GAPDH increased significantly in response to Flv treatment. The 0 h time point was normalized to 1. Values shown are the mean±S.D. (*P<0.05, Student's *t-*test; *n*=3). (**c**) Sig-1R and *β*-actin mRNA expression in Neuro2a cells treated with 10 *μ*g/ml Flv was measured by semiquantitative RT-PCR over the time course shown in the figure. The Sig-1R mRNA level increased by ∼3.8-fold after Flv treatment for 24 h. (**d)** Sig-1R and *β*-actin mRNA expression in Neuro2a cells treated with 10 *μ*g/ml Flv was determined by quantitative RT-PCR over the time course shown in the figure. The Sig-1R mRNA level increased by 32% and 28% after Flv treatment for 12 h and 24 h, respectively. The 0 h time point was normalized to 1. Values are the mean±S.D. (*P<0.05, Student's *t*-test; *n*=3). (**e**) The reporter assay system shown in the figure was constructed. The Sig-1R promoter region (-582 to -156) was fused to a firefly luciferase plasmid (pGL4.12[luc2CP]) and relative luciferase activities were measured. Luciferase activity increased significantly over the time course shown in response to treatment with 10 *μ*g/ml Flv. Values are the mean±S.D. (*P<0.05, Student's *t-*test; *n*=3)

The effect of Flv on Sig-1R expression was investigated by immunoblot analysis of Sig-1R protein expression in Neuro2a cells following the addition of Flv. A time course analysis (Figures 1a and b) demonstrated that Sig-1R protein expression increased in response to Flv treatment. Expression increased within 6 h and by ∼2.7-fold after 24 h.

Changes in Sig-1R expression at the transcriptional level in response to Flv treatment were investigated. Neuro2a cells were treated with Flv for 2–24 h. mRNA was extracted, and semiquantitative and quantitative reverse transcription (RT)-PCR were performed (Figures 1c and d). The Sig-1R mRNA level increased by 32 and 28% after Flv treatment for 12 h and 24 h, respectively. In comparison, no change was observed in the amount of *β*-actin mRNA, indicating that Flv induced Sig-1R expression at the transcriptional level.

A reporter assay system was constructed in which the Sig-1R 5′ upstream region (−582 to −156) containing the promoter was fused to a firefly luciferase plasmid (pGL4.12 [luc2CP]) (Figure 1e). At 48 h after transfection with the reporter plasmid, HEK293 cells were treated with Flv for different periods. The relative luciferase activity was measured using a dual-luciferase reporter assay system with firefly (*Photinus pyralis*) luciferase activity as the test reporter and sea pansy (*Renilla reniformis*) luciferase activity as the internal control. A standardized value for luciferase activity was calculated (Figure 1e). The relative luciferase activity in the Flv-treated cells after 24 h was ∼2.3-fold higher than that in the untreated cells. These results are consistent with the immunoblot analysis and semiquantitative RT-PCR results. On the other hand, the localization of Sig-1R protein, which was predominantly expressed in the ER, did not change after 24-h treatment of Flv (Supplementary Figure 1).

### Flv-induced Sig-1R expression is mediated by the transcription factor ATF4

**Figure 2:**
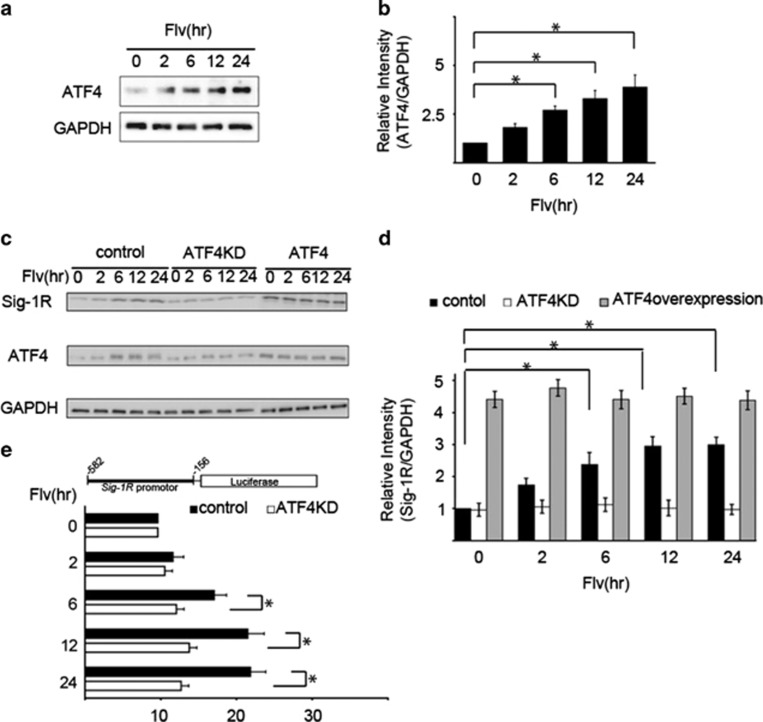
Flv-induced Sig-1R expression is mediated by the transcription factor ATF4. (**a**) Flv (10 *μ*g/ml) was added to Neuro2a cells, and the expression of ATF4 and GAPDH was measured over the time course shown. (**b**) The amount of ATF4 and GAPDH protein in (**a**) was quantified with densitometry. Relative intensity of ATF4/GAPDH increased significantly in response to Flv treatment. The 0 h time point was normalized to 1. Values are the mean±S.D. (*P<0.05, Student's *t*-test; *n*=3). (**c**) Flv (10 *μ*g/ml) was added to HEK293 cells after ATF4 knockdown (ATF4KD), ATF4 overexpression (ATF4), or mock transfection (control). Sig-1R expression was monitored over the time course shown in the figure. In ATF4KD cells, an increase in Sig-1R protein expression was not observed even with Flv treatment. (**d**) The amount of Sig-1R protein from the samples in panel (**c**) was quantified with densitometry. Mock-transfected HEK293 cells (control), ATF4KD, and ATF4 HEK293 cells are indicated by black, white, and gray bars, respectively. The 0 h time point was normalized to 1. Values are the mean±S.D. (*P<0.05, Student's *t*-test; *n*=3). (**e**) The reporter assay system shown in the figure was constructed. The Sig-1R promoter region (-582 to -156) was fused to a firefly luciferase plasmid (pGL4.12[luc2CP]) and transfected into HEK293 cells. After 48 h, control cells (control), cells transfected with control shRNA plasmids, and cells transfected with the ATF4-shRNA-pSuper plasmid (ATF4KD) were treated with 10 *μ*g/ml of Flv over the time course shown in the figure. Relative luciferase activities were measured. Sig-1R expression was not significantly induced by Flv treatment in ATF4 knockdown cells. Values are the mean±S.D. (*P<0.05, Student's *t*-test; *n*=3)

We previously demonstrated that the increase in Sig-1R expression in response to ER stress is mediated by the PERK pathway, one of the cellular responses to ER stress.^9^ Therefore, we investigated whether this mechanism was involved in the Flv-induced expression of Sig-1R by examining changes in ATF4 expression in cells treated with Flv. In Neuro2a cells treated with Flv for different periods, ATF4 protein expression increased (Figures 2a and b). On the basis of these results, we hypothesized that the increase in Sig-1R expression in response to Flv treatment was due to transcriptional activation by ATF4. To examine this premise, we investigated the effect of ATF4 knockdown on Sig-1R expression. HEK293 cells were transfected over 48 h with ATF4-shRNA-pSuper plasmids to knockdown ATF4, control shRNA plasmids, or plasmids incorporating full-length ATF4 to overexpress ATF4. Following treatment with Flv, protein expression was examined by immunoblot analysis (Figures 2c and d). ATF4 and Sig-1R expression increased in both control and ATF4-overexpressing cells treated with Flv. In comparison, in ATF4 knockdown cells, no increase in Sig-1R protein expression was observed even with Flv treatment. These results demonstrate that Flv-induced Sig-1R expression is mediated by ATF4.

The role of ATF4 was further scrutinized using a reporter assay system, in which the Sig-1R gene promoter region (−582–−156) was fused to a firefly luciferase plasmid (pGL4.12 [luc2CP]), as described above. HEK293 cells were transfected with control shRNA plasmids and ATF4-shRNA-pSuper plasmids (ATF4KD). After 48 h, Flv was administered for different periods, and the relative luciferase activities were measured using the dual-luciferase reporter assay system (Figure 2e). Flv treatment induced Sig-1R expression in control cells. In contrast, the expression of Sig-1R in response to Flv treatment was reduced in ATF4KD cells. Taken together, the results indicate that ATF4 is required for the activation of Sig-1R transcription.

### Flv induces Sig-1R expression without activating the unfolded protein response (UPR) during ER stress

**Figure 3:**
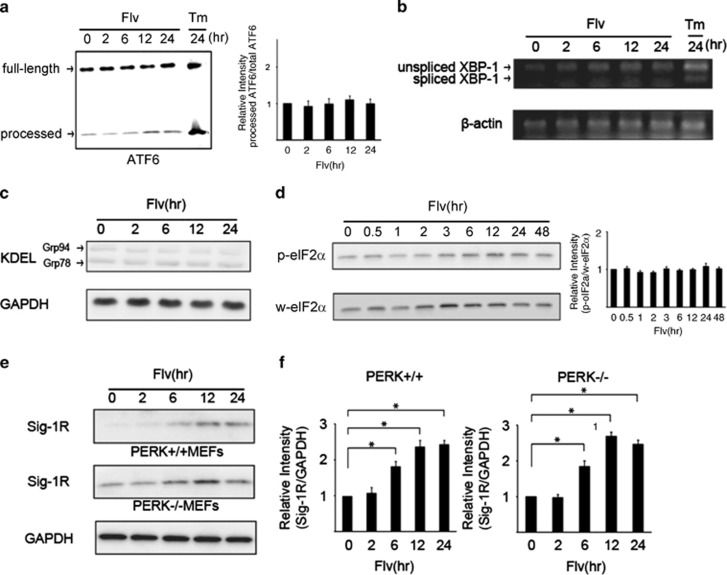
Flv induces Sig-1R expression without activating the unfolded protein response (UPR) during ER stress. (**a**) Flv (10 *μ*g/ml) or tunicamycin (Tm; 1 *μ*g/ml) was added to Neuro2a cells, and changes in ATF6*α* processing were monitored over the time course shown in the figure. The amount of processed ATF6*α* protein increased with Tm treatment but not with Flv treatment. Processed ATF6*α*/total ATF6*α* was measured with densitometry. The results are shown in the bar graph. The 0 h time point was normalized to 1. Values are the mean±S.D. (*P<0.05, Student's *t*-test; *n*=3). (**b**) Flv (10 *μ*g/ml) or Tm (1 *μ*g/ml) was added to Neuro2a cells, and changes in XBP-1 splicing were measured by semiquantitative RT-PCR over the time course shown in the figure. XBP-1 splicing was observed with Tm treatment but not with Flv treatment. (**c**) Flv (10 *μ*g/ml) was added to Neuro2a cells, and changes in the expression of GRP78/BiP and GRP94, which react with anti-KDEL antibodies, were monitored over the time course shown in the figure. No change in the expression of GRP78/BiP or GRP94 as a result of Flv treatment was observed. (**d**) Flv (10 *μ*g/ml) was added to Neuro2a cells, and the expression of total eIF2*α* and phosphorylated eIF2*α* was measured over the time course shown in the figure. No change in the amount of total eIF2*α* (w-eIF2*α*) or phosphorylated eIF2*α* (p-eIF2*α*) as a result of Flv treatment was observed. p-eIF2*α*/w-eIF2*α* was measured with densitometry. The results are shown in the bar graph. The 0 h time point was normalized to 1. Values are the mean±S.D. (*P<0.05, Student's *t*-test; *n*=3). (**e**) Mouse embryonic fibroblasts (MEFs) from PERK wild-type (PERK^+/+^) and PERK knockout (PERK^−/−^) mice were treated with 10 *μ*g/ml of Flv. The expression of Sig-1R and GAPDH was monitored over the time course shown in the figure. Flv treatment induced Sig-1R expression in MEFs from PERK^+/+^ and PERK^−/−^ animals. (**f**) Densitometry analysis of (**e**) showed that relative intensity of Sig-1R/GAPDH increased significantly in both PERK^+/+^ and PERK^−/−^ MEFs in response to Flv treatment. Left graph, PERK^+/+^ MEFs; right graph, PERK^−/−^ MEFs. Values are the mean±S.D. (*P<0.05, Student's *t-*test; *n*=3)

ATF6 and XBP-1 are important transcription factors for the expression of genes related to the UPR caused by ER stress. Therefore, the effect of Flv on the expression of these transcription factors was investigated. Flv or tunicamycin (Tm) was added to Neuro2a cells, and changes in ATF6*α* processing and XBP-1 splicing were assessed. Neither ATF6*α* processing nor XBP-1 splicing changed after Flv treatment, whereas Tm promoted ATF6*α* processing and XBP-1 splicing (Figures 3a and b).

We examined the effect of Flv on the expression of GRP78/BiP or GRP94,^11^ a molecular chaperone that promotes or corrects the folding of unfolded proteins that accumulate in the ER. These chaperones are parts of the UPR during the ER stress response. Flv was added to Neuro2a cells, and changes in the expression of GRP78 and GRP94 were assessed over time. Protein levels were measured by immunoblotting with an anti-KDEL antibody, which recognizes the highly conserved Lys–Asp–Glu–Leu sequence in both proteins. Changes in GRP78 and GRP94 protein expression after Flv treatment were not detected (Figure 3c), indicating that Flv treatment did not induce ER stress.

However, according to our previous report, it is possible that Flv induces Sig-1R expression through the PERK pathway. Thus, we monitored changes in the phosphorylation of eIF2*α* over time in cells treated with Flv. No significant change in the eIF2*α* phosphorylation level was observed in Neuro2a cells treated with Flv (Figure 3d).

We examined whether the Flv-induced expression of Sig-1R occurred through the PERK pathway using mouse embryonic fibroblasts (MEFs) from PERK wild-type (PERK^+/+^) and knockout (PERK^−/−^) mice. PERK^+/+^ and PERK^−/−^ MEFs were treated with Flv for various times, and changes in the expression of Sig-1R were assessed by immunoblot analysis. The expression of Sig-1R protein increased in both MEFs from PERK^+/+^ and PERK^−/−^ mice (Figures 3e and f), indicating that the Flv-mediated induction of Sig-1R protein expression was independent of the PERK pathway.

### Flv induces ATF4 via Sig-1R

**Figure 4:**
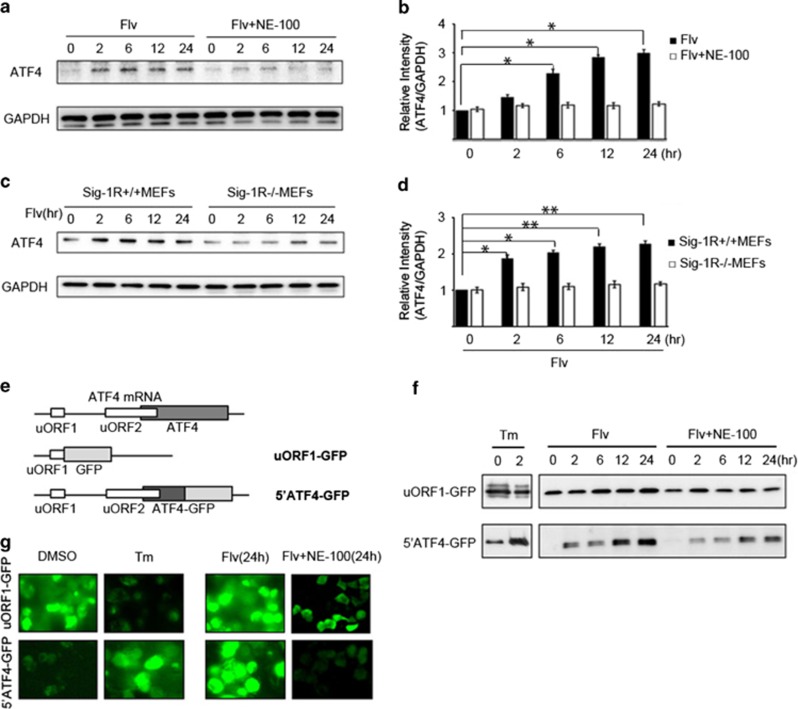
Flv induces ATF4 via Sig-1R. (**a**) Neuro2a cells were treated with Flv (10 *μ*g/ml) or Flv (10 *μ*g/ml) and NE-100 (NE-100a Sig-1R antagonist; 1 *μ*g/ml). The expression of ATF4 and GAPDH was monitored over the time course shown in the figure. (**b**) The amount of ATF4 and GAPDH in (**a**) was quantified with densitometry. Relative intensity of ATF4/GAPDH in cells treated with Flv+NE-100 was significantly suppressed compared with in cells treated with Flv alone. The 0 h time point was normalized to 1. Values are the mean±S.D. (*P<0.05, Student's *t*-test; *n*=3). (**c**) Flv (10 *μ*g/ml) was added to Sig-1R^+/+^ MEFs and Sig-1R^−/−^ MEFs. Changes in ATF4 and GAPDH expression were monitored over the time course shown. (**d**) The amount of ATF4 and GAPDH in (**c**) was quantified with densitometry. Relative intensity of ATF4/GAPDH showed that ATF4 induction in Sig-1R+/+ MEFs treated with Flv was not observed in Sig-1R^−/−^ MEFs treated with Flv. The 0 h time point was normalized to 1. Values are the mean±S.D. (*P<0.05, **P<0.01, Student's *t*-test; *n*=3). (**e**) To investigate the regulation of ATF4 translation by Flv, we used the reporter assay system shown in the figure.^11^ One construct contained ATF4 uORF1 fused with a GFP coding region (uORF1-GFP). A second construct contained a fusion of ATF4 uORF1, uORF2, the ATF4 N terminus, and a GFP coding region (5′ATF4-GFP). (**f**) HEK293 cells were transfected with the uORF1-GFP reporter construct or the 5′ATF4-GFP reporter construct shown in (**e**). Thereafter, Tm (1 *μ*g/ml), Flv (10 *μ*g/ml), or Flv (10 *μ*g/ml) and NE-100 (1 *μ*g/ml) were added. Protein expression over the time course shown in the figure was assessed by immunoblot analysis. Changes in GFP expression were monitored. With Tm treatment, GFP expression in uORF1-GFP cells decreased, whereas GFP expression in 5′ATF4-GFP cells increased. On the other hand, with Flv treatment, GFP expression increased in both uORF1-GFP cells and 5′ATF4-GFP cells. The increase in GFP expression observed in uORF1-GFP and 5′ATF4-GFP cells treated with Flv was suppressed when the cells were treated with Flv+NE-100. (**g**) Cell transfected and treated as described for (**f**) were observed by fluorescence microscopy. In DMSO-treated cells, uORF1-GFP expression was observed, but 5′ATF4-GFP expression was not detected. In contrast, in Tm-treated cells, uORF1-GFP expression decreased, and 5′ATF4-GFP expression increased, suggesting that ER stress selectively promoted the translation of ATF4. In contrast, increased GFP expression was observed in both uORF1-GFPand 5′ATF4-GFP-transfected cells after Flv treatment (24 h). In both cell lines, the increase in GFP expression was suppressed by treatment with Flv+NE-100 (24 h), consistent with the results of the immunoblot analysis in (**f**)

We investigated the molecular mechanism of Flv's effect on ATF4. Neuro2a cells were treated with Flv, alone or in combination with NE-100, a Sig-1R antagonist, for various times. The increase in ATF4 protein expression observed in cells treated with Flv alone was sharply attenuated in cells treated with Flv+NE-100 (Figures 4a and b). Furthermore, we examined whether Flv induced ATF4 expression in MEFs from Sig-1R knockout (Sig-1R^−/−^) mice. Whereas Flv treatment increased ATF4 expression in MEFs from Sig-1R^+/+^ mice, Flv treatment did not increase ATF4 protein expression in MEFs from Sig-1R^−/−^ mice (Figures 4c and d).

In ER stress, activation of PERK leads to the phosphorylation of the translation initiation factor eIF2*α* and consequently to translational attenuation. In contrast, in ATF4 mRNA, when translation is attenuated, ribosomes skip the ordinal-acting element uORF1 at the 5′ end because of eIF2*α* phosphorylation by PERK. When this occurs, ribosomes reinitiate translation downstream at uORF2, which is the actual ATF4 coding region.^12, 13^ To investigate the translational regulation of ATF4 by Flv, we used a construct in which ATF4 uORF1 and a green fluorescent protein (GFP) coding region were fused (uORF1-GFP) and a construct in which ATF4 uORF1, uORF2, the ATF4 N terminus, and a GFP coding region (5′ATF4-GFP) were fused (supplied by D. Ron, Figure 4e). HEK293 cells were transfected with each of these constructs. After 48 h, cells were treated with Tm or Flv for various times, and changes in GFP expression were determined by immunoblot analysis. With Tm treatment, GFP expression in cells transfected with uORF1-GFP decreased. In contrast, GFP expression increased in cells expressing the 5′ATF4-GFP fusion construct. Intriguingly, with Flv treatment, GFP expression increased in cells transfected with uORF1-GFP and also in cells transfected with 5′ATF4-GFP. In both transfected cell types, the increase in GFP expression induced by Flv treatment was suppressed when the cells were treated with Flv+NE-100 (Figure 4f).

HEK293 cells transfected with the uORF1-GFP and 5′ATF4-GFP reporter constructs were treated with dimethyl sulfoxide (DMSO) (control), Tm, Flv, or Flv+NE-100. After 24 h, GFP expression in cells was observed by fluorescence microscopy to monitor the changes in ATF4 translation. As shown in Figure 4g, fluorescence was observed in DMSO-treated cells transfected with uORF1-GFP, but not in cells transfected with 5′ATF4-GFP. Tm treatment decreased uORF1-GFP expression and increased 5′ATF4-GFP expression, suggesting that translation of ATF4 was selectively promoted under ER stress. In contrast, Flv treatment increased GFP expression in both uORF1-GFP- and 5′ATF4-GFP-transfected cells, but the increase was suppressed by the addition of NE-100, consistent with the results of the immunoblot analysis. From these results, we hypothesized that Flv increases ATF4 protein expression by promoting the Sig-1R signal.

### Flv suppresses ER stress-mediated apoptosis

**Figure 5:**
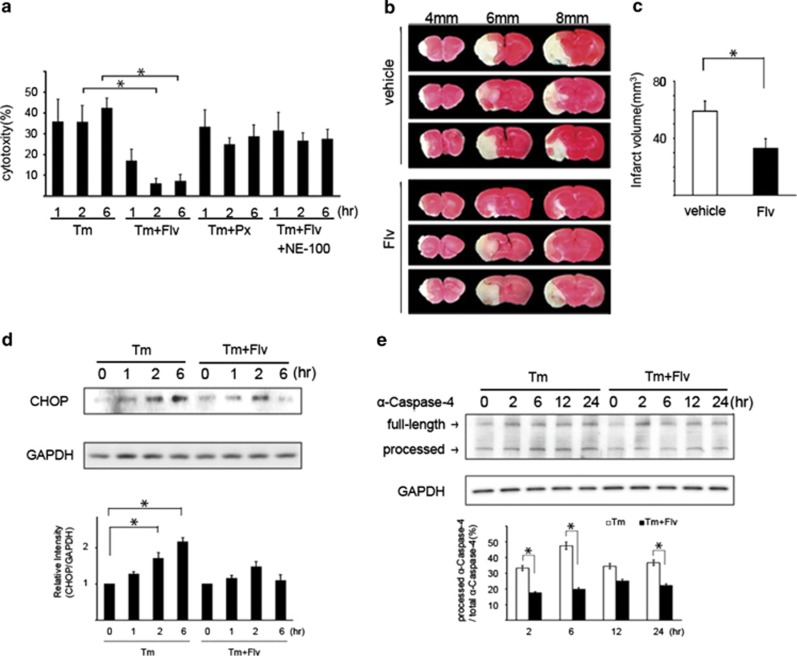
Flv suppresses ER stress-mediated apoptosis. (**a**) Tm, Tm+Flv, Tm+Paroxetine (Px), or Tm+Flv+NE-100 were added to Neuro2a cells. The free LDH activity was measured over the time course shown in the figure, and cytotoxicities were compared. At the times shown in the figure, cells were treated with 10 *μ*g/ml of Flv, 1 *μ*g/ml of Tm, 1 *μ*g/ml of Px, and 1 *μ*g/ml of NE-100. The cytotoxicity induced by Tm+Flv treatment was significantly lower than that induced by treatment with Tm alone. No change in cytotoxicity was observed with Tm+Px treatment. With Tm+Flv+NE-100 treatment, the effect of Flv was negated. Values are the mean±S.D. (*P<0.05, Student's *t*-test; *n*=3). (**b**) Flv administration reduces the area and volume of the cerebral infarct in mice subjected to middle cerebral artery occlusion (MCAO). MCAO was performed in mice administered an intraperitoneal injection of vehicle (20 mg/kg, *n*=9) or Flv (20 mg/kg, *n*=9). Cerebral cortex slices were prepared 24 h later. (The forebrain was divided into five coronal 2-mm sections). The slices were stained with TTC, and the areas and volumes of the respective cerebral infarct lesions were compared. The photograph in the figure shows slices 4, 6, and 8 mm from the front of the brain. A significant reduction in the area of the cerebral infarct lesion was observed in Flv-treated mice. Three upper images: representative images of cortex slices from vehicle-treated mice. Three lower images: representative images of cortex slices from Flv-treated mice. (**c**) MCAO was performed in mice treated with Flv or vehicle. Brain slices from mice in each group were stained with TTC. The area of the cerebral infarct lesion was measured with image analysis software from a photograph of the cut surface taken with a digital camera. Using reported methods, the volume of the cerebral infarct lesion was calculated from the area of the cerebral infarct lesion measured in (**b**).^15, 16^ The volume of the cerebral infarct lesion in mice treated with Flv was significantly reduced. Values are the mean±S.D. (*P<0.05, Student's *t-*test; *n*=3). (**d**) Tm (1 *μ*g/ml) or Tm (1 *μ*g/ml) and Flv (10 *μ*g/ml) were added to Neuro2a cells, and CHOP expression was monitored over the time course as shown in the figure. A significant reduction in CHOP expression was observed at 2 h and 6 h with Tm+Flv treatment when compared with expression after treatment with Tm alone. CHOP expression was measured with densitometry. The results are shown in the bar graph. The 0 h time point was normalized to 1. Values are the mean±S.D. (*P<0.05, Student's *t*-test; *n*=3). (**e**) Tm (1 *μ*g/ml) or Tm (1 *μ*g/ml) and Flv (10 *μ*g/ml) were added to HEK293 cells, and *α*-caspase-4 processing was monitored over the time course shown in the figure. Processed *α*-caspase-4/total *α*-caspase-4 was measured with densitometry. The results are shown in the bar graph. With the Tm+Flv treatment, *α*-caspase-4 processing at 2, 6, and 24 h was significantly reduced compared with processing after treatment with Tm alone, indicating that Flv treatment reduced *α*-caspase-4 activation. Values are the mean±S.D. (*P<0.05, Student's *t*-test; *n*=3)

Because our previous report showed that the induction of Sig-1R under ER stress suppresses ER stress-mediated apoptosis,^9^ it is interesting whether the induction of Sig-1R by Flv has same effect. The cytotoxicity resulting from ER stress was evaluated with lactate dehydrogenase (LDH) assays. Tm, Tm+Flv, Tm+paroxetine (Px), or Tm+Flv+NE-100 were added to Neuro2a cells. Px is another typical SSRI. Free LDH activity in each treatment condition was measured over time to assess cytotoxicity. Tm+Flv treatment reduced cytotoxicity, when compared with treatment with Tm alone. In contrast, with Tm+Px treatment, no reduction in cytotoxicity was detected. Tm+Flv+NE-100 treatment negated the effect of Flv. These observations indicate that Flv suppresses ER stress-mediated apoptosis through Sig-1R (Figure 5a).

Because cerebral ischemia induces ER stress,^14^ a middle cerebral artery occlusion (MCAO) model was used as an *in vivo* experimental system to validate the effects of Flv. MCAO was performed in groups of mice (*n*=9) following intraperitoneal injection of vehicle (20 mg/kg) or Flv (20 mg/kg). Cerebral cortex slices were sampled from each of the mice 24 h after MCAO. The forebrain was divided into five coronal 2-mm sections. The slices were stained with TTC, and the area of the cerebral infarct lesion was measured with image analysis software from a photograph of the cut surface taken with a digital camera. When the areas of the infarct lesions were compared, the area of the cerebral infarct lesion in the Flv-treated mice was smaller than that in the vehicle-treated mice (Figure 5b). The volume of the cerebral infarct lesion was calculated from the area of the cerebral infarct lesion using reported methods.^15, 16^ The volume of the cerebral infarct lesion in mice administered Flv was smaller than that in vehicle-treated mice (Figure 5c).

The expression of C/EBP homologous protein (CHOP) and processing of human *α*-caspase-4, which is identical to murine caspase-12, are indicators of apoptosis induced by ER stress.^17, 18^ We investigated whether Flv attenuated the ER stress-mediated apoptosis pathway by monitoring CHOP expression and *α*-caspase-4 processing. After the addition of Tm or Tm+Flv to Neuro2a cells, CHOP expression and *α*-caspase-4 processing were assessed over time. CHOP expression was reduced at 2 and 6 h in cells treated with Tm+Flv treatment, relative to CHOP expression in cells treated with Tm alone (Figure 5d). Tm+Flv treatment to HEK293 cells also reduced *α*-caspase-4 processing, when compared with treatment with Tm alone (Figure 5e). It suggests that Flv prevents the apoptosis by ER stress in human cells, which express caspase-4 substituting for caspase-12. These results indicate that Flv can suppress the ER stress-mediated cell death signal.

### Flv induces neuroprotection by increasing Sig-1R protein expression

**Figure 6:**
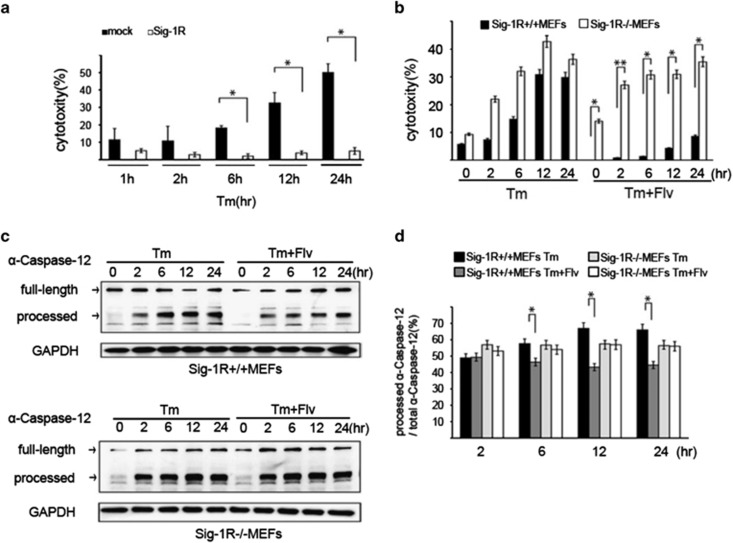
Flv induces neuroprotection by increasing Sig-1R protein expression. (**a**) Tm (1 *μ*g/ml) was added to mock-transfected HEK293 cells and Sig-1R-overexpressing HEK293 cells. For cytotoxicity comparisons, the free LDH activity was measured over the time course shown in the figure. Cytotoxicity was significantly reduced at 6, 12, and 24 h in Sig-1R expressing cells. Values are the mean±S.D. (*P<0.05, Student's *t*-test; *n*=3). (**b**) Tm (1 *μ*g/ml) or Tm (1 *μ*g/ml) and Flv (10 *μ*g/ml) were added to Sig-1R^+/+^ and Sig-1R^−/−^ MEFs. The free LDH activity in each treatment condition was measured over the time course shown in the figure, and the cytotoxicities were compared. After Flv treatment, a significant reduction in toxicity was observed in Sig-1R^+/+^ MEFs but not in Sig-1R^−/−^ MEFs. Values are the mean±S.D. (*P<0.05, Student's *t*-test; *n*=3). (**c**) In the upper figure, Tm (1 *μ*g/ml) or Tm (1 *μ*g/ml) and Flv (10 *μ*g/ml) were added to Sig-1R^+/+^ MEFs, and caspase-12 processing was monitored over the time course shown. Caspase-12 processing was significantly reduced with Tm+Flv treatment compared with processing after treatment with Tm alone, indicating that Flv suppressed the induction of cell death. In the lower figure, Tm (1 *μ*g/ml) or Tm (1 *μ*g/ml) and Flv (10 *μ*g/ml) were added to Sig-1R^−/−^ MEFs, and caspase-12 processing was monitored over the time course shown. Caspase-12 processing in Sig-1R-deficient cells was not affected by the addition of Flv. (**d**) Processed caspase-12/total caspase-12 in (**c**) was measured with densitometry. Black bar: Sig-1R^+/+^ MEFs treated with Tm; dark gray bar: Sig-1R^+/+^ MEFs treated with Tm+Flv; light gray bar: Sig-1R^−/−^ MEFs treated with Tm; white bar: Sig-1R^−/−^ MEFs treated with Tm+Flv. Processed caspase-12 was significantly reduced by Flv treatment in Sig-1R^+/+^ MEF cells but not in Sig-1R^−/−^ MEF cells, demonstrating that the ability of Flv to suppress cell death induction was lost in the absence of Sig-1R. Values are the mean±S.D. (*P<0.05, Student's *t*-test; *n*=3)

We examined whether the anti-ER stress effect observed after Flv treatment was mediated by Sig-1R expression. Tm was added to mock-transfected HEK293 cells and Sig-1R-overexpressing HEK293 cells. LDH activity was measured over time to assess cytotoxicity. A significant reduction in the cytotoxicity induced by Tm treatment was observed at 6, 12, and 24 h in Sig-1R-expressing cells (Figure 6a). Subsequently, Tm or Tm+Flv were added to MEFs from Sig-1R^+/+^ and Sig-1R^−/−^ mice, and LDH assays were performed. In Sig-1R^+/+^ MEFs treated with Flv, a significant reduction in the cytotoxicity induced by Tm treatment was observed. In contrast, treatment of Sig-1R^−/−^ MEFs with Flv had no effect on cytotoxicity (Figure 6b).

Caspase-12 processing is an indicator of apoptosis induced by ER stress.^18^ Tm or Tm+Flv were added to Sig-1R^+/+^ MEF cells, and caspase-12 processing was monitored over time. Caspase-12 processing was significantly reduced in the presence of Flv (Figures 6c and d), indicating that Flv suppressed the induction of cell death. In comparison, there was no difference in caspase-12 processing in cells treated with Tm and cells treated with Tm+Flv (Figures 6c and d). These results demonstrate that Flv does not induce neuroprotection in the absence of Sig-1R. These results indicate that resistance to ER stress was due to increased Sig-1R expression.

## Discussion

The results of this study clearly show that Flv, via Sig-1R signaling, induces Sig-1R expression by increasing ATF4 translation, without involving the PERK pathway. In addition, Flv-mediated upregulation of Sig-1R suppresses ER stress-mediated apoptosis to produce a neuroprotective effect. The data strongly suggest that this molecular mechanism offers a new treatment target for diseases thought to be caused by ER stress such as neurodegenerative diseases^19, 20^ and for diseases associated with reduced Sig-1R levels such as schizophrenia and depression.^5, 6^

Questions remain regarding the molecular mechanisms by which Flv induces Sig-1R via a translation of ATF4 without the involvement of the whole PERK pathway. How does activated Sig-1R interact with ATF4, and does it affect the translational regulation of ATF4? In the absence of ER stress, the eIF2-GTP-methionyl start tRNA (Met-tRNAi^Met^) ternary complex is abundant. In this setting, after translation from uORF1 at the 5′end of the ATF4 mRNA, ribosomes dissociate from the mRNA. Accordingly, ATF4 is not translated. However, upon induction of ER stress and eIF2*α* phosphorylation, recycling of GTP-bound eIF2*α* is blocked because phosphorylated eIF2*α* binds eIF2B with high affinity and inhibits its activity.^21^ Consequently, the intracellular concentration of the ternary complex decreases, and the translation start efficiency is reduced. Furthermore, because ribosomes remain bound to mRNA, downstream movement continues. Initiation factors do not bind to ternary complexes until they arrive at uORF2 in the downstream region of ATF4, resulting in translation of ATF4.^12, 13^ In the present study, we showed that translation from uORF1 and uORF2 occurs at the same time when Sig-1R is induced by Flv. We also showed that Flv induces ATF4 by an activation of Sig-1R. Additional verification of this result is needed, but we think that activation of Sig-1R may affect the formation of eIF2-GTP-Met-tRNAi^Met^.

A relationship between Sig-1R gene polymorphisms, such as GC-241-240TT and Gln2Pro, and schizophrenia has been reported.^22^ Other reports have demonstrated that ATF4 gene polymorphism is involved in schizophrenia.^23^ In *in vitro* studies, we have shown that the PERK pathway induces Sig-1R expression during the ER stress response.^9^ Moreover, the mechanism by which the PERK pathway induces Sig-1R expression is impeded in schizophrenia when the GC-241-240TT and T-485A polymorphisms are present.^9^ In schizophrenia patients, the induction of Sig-1R expression by ATF4, which is thought to function in the UPR pathway, may be impaired for some reason. We speculate that insufficiency in the ATF4-Sig-1R pathway causes psychiatric symptoms in schizophrenia patients. Our studies have demonstrated that this pathway is important in suppressing ER stress, and we consider this to be a fourth pathway in the UPR, which consists of translational attenuation, transcriptional induction, and ER-associated degradation (ERAD). There have been reports that symptoms and cognitive function improve in schizophrenia patients administered Flv.^24^ This might be explained by the Flv-mediated activation of the ATF4-Sig-1R pathway.

Some studies have indicated that increased expression of Sig-1R has a physiological function. Nerve growth factor receptor signal transmission and nerve growth factor-induced neurite elongation as a result of Sig-1R overexpression in cells have been observed.^25^ Moreover, although the mechanism has not been fully elucidated, Flv, via Sig-1R, promotes neurite elongation.^25^ Therefore, Sig-1R expression may have an important role in synapse formation and plasticity. On the other hand, there are reports that stimulation of brain-derived neurotrophic factor (BDNF) promotes the translocation of the ER stress molecule XBP-1 from neurites to the nucleus, resulting in increased expression of genes needed for neurite elongation.^26^ It is possible that cellular responses to ER stress contribute not only to pathological conditions, but also to normal neural development. Because of these findings, further study is required to determine what effect the mitigation of ER stress by Flv-induced Sig-1R expression has on the downstream signaling of nerve growth factor receptor. Similarly, additional research could elucidate the effect of Flv on neurite development and the physiological role of ER stress in synapse formation and plasticity.

Recently, the involvement of ER stress in various diseases, such as diabetes, cancer, and ischemic cardiac disease, has been reported.^27, 28, 29^ With regard to neurodegenerative diseases, a series of reports have described the involvement of ER stress in Alzheimer's disease,^28, 29^ Parkinson's disease,^30^ and polyglutamine diseases.^19^ We have described the relationship between presenilin 1 and ER stress.^31, 32^ Given these findings, drugs that suppress ER stress may be valuable new targets in the treatment of these diseases. We are currently developing a novel compound referred to as molecular chaperone derivative BiP inducer X (BIX). The addition of BIX to nerve cells or the intraventricular administration of BIX to mice suppresses apoptosis caused by ER stress.^20^ With this study, we add Flv to the list of potential drugs against ER stress. Importantly, because Flv is commonly used in clinical practice, it will face fewer clinical testing hurdles than newly developed drugs. Therefore, in future research, we anticipate clinical testing of Flv in several diseases brought on by ER stress, including ischemic diseases such as cerebral infarction.

In conclusion, we have shown *in vitro* and *in vivo* that Flv, via activation of Sig-1R, upregulates Sig-1R expression by increasing ATF4 translation and thereby inhibits cell death resulting from ER stress. To facilitate the clinical application of newly developed drugs that mitigate ER stress, including Flv, basic research using Sig-1R knockout mice and postmortem brain tissue will be necessary. Furthermore, positron emission tomography studies^33^ will be valuable for elucidating Flv activity because they enable quantitative measurement of Sig-1R in the brains of living patients and monitoring of the therapeutic dose required to induce the expression of Sig-1R and other effector proteins.

## Materials and Methods

### Cell culture and chemicals

HEK293 cells and mouse Neuro2a cells were cultured in DMEM (Invitrogen, Carlsbad, CA) supplemented with 10% fetal bovine serum (FBS; JRH Biosciences, Leneza, KS, USA) at a density of 1 × 10^6^ cells/well in 6-well plates. PERK^+/+^ (wild-type) or PERK^−/−^ (knockout) MEFs were cultured in DMEM plus 10% FBS. Sig-1R^+/+^ or Sig-1R^−/−^ MEFs were also cultured in DMEM plus 10% FBS. Tm, Flv, Px, 2,3,5-triphenyltetrazolium chloride (TTC), *N,N*-dipropyl-2-[4-methoxy-3-(2-phenylethoxy)phenyl]-ethylamine monohydrochloride (NE-100), and DMSO were purchased from Sigma-Aldrich (St. Louis, MO, USA). Cells were transfected with plasmids using calcium phosphate reagent (Invitrogen).

### Immunoblotting

For immunoblotting, cells were lysed with RIPA buffer (Thermo Scientific, Fremont, CA, USA) supplemented with a protease inhibitor cocktail and phosphatase inhibitor cocktail (Roche, Mannheim, Germany). The lysates were incubated on ice for 15 min. After centrifugation at 13 000 × *g* for 20 min, soluble proteins in the extracts were quantified. An equal amount of extract (20 *μ*g) from each sample was electrophoresed in a sodium dodecyl sulfate-polyacrylamide gel (Biocraft, Tokyo, Japan) and then transferred to a polyvinylidene fluoride membrane (Immobilon-P; Millipore, Billerica, MA, USA) for immunoblotting. Antibodies against the following proteins were used at the dilutions indicated: Sig-1R (ab53852; 1:250; Abcam, Cambridge, UK), eukaryotic initiation factor-2*α* (eIF2*α*) (#9722, 1:1000; Cell Signaling Technology, Danvers, MA, USA), phospho-eIF2*α* (#9721; 1:1000; Cell Signaling Technology), activating transcription factor 4 (ATF4) (sc-22800; 1:1000; Santa Cruz Biotechnology, Dallas, TX, USA), ATF6*α* (sc-22799; 1:1000; Santa Cruz Biotechnology), X-box binding protein 1 (XBP-1) (sc-7160; 1:1000; Santa Cruz Biotechnology), KDEL ER marker (sc-58774; 1:1000; Santa Cruz Biotechnology), GFP (D153-9; 1:5000; MBL, Woburn, MA, USA), glyceraldehyde 3-phosphate dehydrogenase (GAPDH) (MA1-10036; 1:2500; Thermo Scientific, Waltham, MA, USA), caspase-4 (M029-3; 1:1000; MBL), caspase-12 (#2202; 1:1000; Cell Signaling Technology), CHOP (sc-7351; 1:1000; Santa Cruz Biotechnology), calnexin (ADI-SPA-860; 1:500; Stressgen, VIC, Canada), and GM130 (#610822; 1:500; BD Transduction Laboratories, Franclin Lakes, NJ, USA). Specific antigen-antibody complexes were visualized using horseradish peroxidase-conjugated secondary antibodies and an enhanced chemiluminescence plus kit (GE Healthcare, Buckinghamshire, UK).

### Plasmids

Plasmids uORF1-GFP and 5′ATF4-GFP were provided by Dr. David Ron (University of Cambridge Metabolic Research Laboratories, Cambridge, UK).

### RNA extraction and semiquantitative RT-PCR

Total RNA was extracted from cells using TRIzol reagent (Invitrogen) according to the manufacturer's protocol. For each extract, the RNA concentration was determined by spectrophotometry at 260 nm, and 2 *μ*g of RNA was used with a PrimeScript II 1st Strand cDNA Synthesis Kit (Takara, Shiga, Japan) for the RT reaction. PCR was performed in 50 *μ*l containing 0.8 *μ*M of each primer, 0.2 mM dNTPs, 1.25 U of Ex-Taq polymerase, and 10 × PCR buffer (Takara). The primer sets used for amplification were as follows: Sig-1R forward, 5′-GCCTTCTCTCGTCTGATCGT-3′ and Sig-1R reverse, 5′-GCCAAAGAGGTAGGTGGTGA-3′ *β*-actin forward, 5′-GTTTGAGACCTCAACACC-3′ and *β*-actin reverse, 5′-GTGGTGGTGAAGCTTAG-3′, X-box binding protein 1 (XBP-1), 5′-TAAGACAGCGCTTGGGGATGG-3′, and 5′-CAGAATCCATGGGGAGATGTT-3′. PCR was performed for 25 cycles of 30 s at 95 °C, 30 s at 60 °C, and 1 min at 72 °C. PCR products were analyzed by agarose gel electrophoresis.

### Quantitative PCR/TaqMan gene expression assay

Quantitative PCR was carried out using the Applied Biosystems (Carlsbad, CA, USA) ViiA 7 Real-Time PCR System, according to the manufacturer's instructions. Primers and probes for quantitative PCR were purchased from Applied Biosystems (Sig-1R, Mm00448086_m1 and *β*-actin, Mm00607939_s1). The cycle conditions for quantitative PCR were 95 °C for 10 min, followed by 40 cycles of 95 °C for 15 s and 60 °C for 1 min.

### Subcellular fractionation

Neuro2a cells in 10-cm diameter dishes were harvested in 1.0 ml of ice-cold homogenization buffer (0.25 M sucrose and 10 mM HEPES, pH 7.4) and homogenized by 10 passages through a 25-G needle. Post-nuclear supernatants were obtained by centrifugation at 9500 × *g* for 10 min at 4 °C, and fractionated by centrifugation through linear gradients of 8–25% iodixanol (OptiPrep; Axis-Shield, Oslo, Norway). A step gradient was created in Seton SW41 centrifuge tubes (#151-514A; Seton, Branford, CT, USA) by loading 8 and 25% iodixanol with a Gradient Master (107-201 M; SK Bio International, Tokyo, Japan). Next, 1600-*μ*l aliquots of the post-nuclear supernatants were layered on top of the gradients and fractionated by centrifugation at 100 000 × *g* for 4 h at 4 °C. After centrifugation, 10 fractions (1 ml each) were collected from the top of each tube using a Gradient Fractionator (152 type; SK Bio International). The subcellular fractions were characterized by probing with antibodies against specific marker proteins for various subcellular compartments, such as calnexin and GM130.

### Knockdown of ATF4

The ATF4-shRNA-pSuper plasmid (Oligoengine, Seattle, WA, USA) was transfected into HEK293 cells to generate ATF4 knockdown cells as previously described.^34^ The following oligonucleotides for knockdown of ATF4 were chosen using GENETYX-MAC software (Genetyx Corporation, Tokyo, Japan): 5′-GATCCCCGCCTAGGTCTCTTAGATGATTCAAGAGATCATCTAAGAGACCTAGGCTTTTTGGAAA-3′ and 5′-AGCTTTTCCAAAAAGCCTAGGTCTCTTAGATGATCTCTTGAATCATCTAAGAGACCTAGGGCGGG-3′. The following oligonucleotides were used for control: 5′-GATCCGUGTGGTCTACAGTACGAATTCAAGAGATTCGTACTGTAGACCACATTTTTTCTCGAGG-3′ and control bottom, 5′-GATCCGTGTGGTCTACAGTACGAATTCAAGAGATTCGTACTGTAGACCACATTTTTTCTCGAGG-3′.

### Luciferase assay

The 5′ upstream region of the human *Sig-1R* gene (from −582 to −156) was subcloned upstream of the *luc2CP* luciferase reporter gene in the pGL4.12 [luc2CP] vector. The reporter plasmids were transfected into HEK293 cells. The *Renilla* luciferase plasmid pRL-TK was co-transfected to correct for transfection efficiency. At 48 h after transfection, luciferase assays were performed using the Dual-Luciferase Reporter Assay System (Promega, Madison, WI, USA) and a Wallac 1420 ARVOsx luminometer (Perkin-Elmer, Waltham, MA, USA).

### Microscopy

The cellular fluorescence of uORF1-GFP and 5′ATF4-GFP was assessed using a BZ-9000 fluorescence microscope (Keyence, Chicago, IL, USA).

### LDH cytotoxicity assay

Cell viability was estimated by the LDH leakage method using a cytotoxicity detection kit (Roche) according to the manufacturer's instructions. LDH activity was measured at optical density 490 nm.

### Focal cerebral ischemia mouse model

The experimental design and procedures for animal studies were conducted in accordance with the US National Institutes of Health Guide for the Care and Use of Laboratory Animals and the Animal Care Guidelines issued by the Animal Experimental Committee of Gifu Pharmaceutical University. Experiments were performed using male ddY mice (4-week old; body weight, 17–23 g; Japan SLC Ltd., Shizuoka, Japan). Animals were housed at 24±2 °C under a 12-h light–dark cycle. Every effort was made to minimize the number of animals used and their suffering. Mice were anesthetized with 2–3% isoflurane and maintained under 1.0–1.5% isoflurane in 70% N_2_O and 30% O_2_ using a facemask. Focal cerebral ischemia was induced by inserting an 8-0 nylon monofilament (Ethicon, Somerville, NJ, USA) coated with silicone hardener mixture (Xantopren; Bayer Dental, Osaka, Japan) via the internal carotid artery, as previously described.^15^ In brief, a coated filament was introduced into the left internal carotid artery through the common carotid artery, then advanced to the origin of the anterior cerebral artery via the internal carotid artery so as to occlude the middle cerebral artery and posterior communicating artery. At the same time, the left common carotid artery was occluded. Anesthesia exposure did not exceed 10 min. After 2 h of occlusion, the animals were re-anesthetized briefly, and reperfusion was initiated by withdrawal of the monofilament. After surgery, the mice were kept for another 24 h in a cage under a heat lamp, which maintained the cage temperature at 29–30 °C. Thereafter, the mice were kept in preoperative conditions (24±2 °C) until sampling. Flv (20 mg/kg in DMSO) or DMSO (vehicle) was administered intraperitoneally 1 h before MCAO and again just before MCAO. At 24 h after MCAO, mice were administered an overdose of pentobarbital sodium and then decapitated. The forebrain was divided into five coronal 2-mm sections using a mouse brain matrix (RBM-2000C; Activational Systems, Warren, MI, USA). These slices were immersed for 20 min in 2% TTC in normal saline at 37 °C, then fixed in 10% phosphate-buffered formalin at 4 °C. TTC reacts with intact mitochondrial respiratory enzymes to generate a bright red color that contrasts with the pale color of the infarct. The caudal face of each slice was photographed. Images of the infarcted areas and volumes (unstained) were recorded using a digital camera (Coolpix 4500; Nikon, Tokyo, Japan) and quantified using ImageJ software (http://rsbweb.nih.gov/ij/). The volume of the infarct region was calculated as described in a previous report.^16^

### Statistical analysis

A two-tailed paired Student's *t*-test was used to determine whether the treatment group was significantly different from the control. A *P-*value <0.05 was considered statistically significant. In the figures, the intensity of each band was quantified using Photoshop software (Adobe, San Jose, CA, USA). All graphical data are shown as the mean ±S.D.

## Abbreviations

Sig-1R: sigma-1 receptor
ER: endoplasmic reticulum
Flv: fluvoxamine
SSRI: selective serotonin reuptake inhibitor
ATF4: activating transcription factor 4
PERK: PKR-like ER kinase
NMDA receptor: *N*-methyl-D-aspartate receptor
BiP: binding immunoglobulin protein
IP3 receptor: inositol 1,4,5-trisphosphate receptor
HEK293 cells: human embryonic kidney 293 cells
shRNA: small hairpin RNA
KD: knockdown
UPRs: unfolded protein responses
ATF6: activating transcription factor 6
XBP-1: X-box binding protein 1
GRP: glucose-regulated protein
KDEL: lysine-aspartic acid-glutamic acid-leucine
eIF2*α*: eukaryotic translation initiation factor 2*α*
MEFs: mouse embryonic fibroblasts
WT: wild type
KO: knockout
mRNA: messenger RNA
ORF: open reading frame
GFP: green fluorescent protein
DMSO: dimethyl sulfoxide
Tm: tunicamycin
CHOP: C/EBP homologous protein
LDH assay: lactate dehydrogenase
Px: paroxetine
NE-100: (N,N-dipropyl-2-[4-methoxy-3-(2-phenylethoxy)phenyl]-ethylamine monohydrochloride
MCAO: middle cerebral artery occulusion
TCC staining: triphenyl tetrazolium chloride staining
BDNF: brain-derived neurotrophic factor
ERAD: ER associated degradation
Met-tRNAi^Met^: eIF2-GTP-methionyl start tRNA
GTP: guanosine triphosphate
PS1: presenilin 1
BIX: BiP inducer X
PET: positron emission tomography
