# Supplementary figures and images for: Fluvoxamine alleviates ER stress via induction of Sigma-1 receptor
Source: Cell Death Dis. 2014 Jul 17;5(7):e1332–. doi: 10.1038/cddis.2014.301 (PMC4123092; doi:10.1038/cddis.2014.301)

TOP

BOTTOM

1 2 3 4 5 6 7 8 9 10 11

calnexin

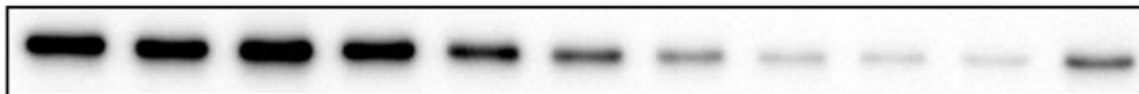

GM130

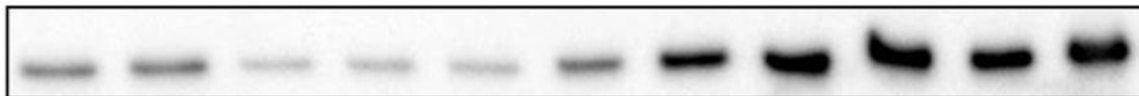Sig-1R  
+DMSO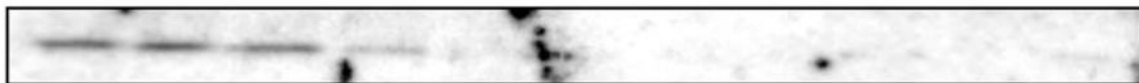Sig-1R  
+Flv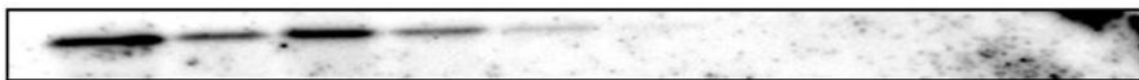

Supplement: Supplementary Figure 1 [file cddis2014301x1.pdf]
