# Supplementary material for: Fluvoxamine alleviates ER stress via induction of Sigma-1 receptor
Source: Cell Death Dis. 2014 Jul 17;5(7):e1332–. doi: 10.1038/cddis.2014.301 (PMC4123092; doi:10.1038/cddis.2014.301)
Supplement: Supplementary Figure 1 Legend [file cddis2014301x2.doc]

**Supplementary Figure Legend**

**Supplemental Fig.1** Subcellular fractionation of neurons treated with fluvoxamine.

Post-nuclear supernatants of Neuro2a cells with or without 24-hour treatment of Flv (10 μg/mL) were fractionated by centrifugation using a linear gradients of 8-25% iodixanol (Optiprep; AXISSHIELD). Western blot shows that a localization of Sig-1R does not change by fluvoxamine treatment. Calnexin and GM130 were used as ER and Golgi maker respectively.
